# Genome Sequence of the Black Yeast-Like Strain Aureobasidium pullulans var. *aubasidani* CBS 100524

**DOI:** 10.1128/MRA.01293-20

**Published:** 2021-03-25

**Authors:** Gabriel A. Vignolle, Robert L. Mach, Astrid R. Mach-Aigner, Christian Derntl

**Affiliations:** a Institute of Chemical, Environmental and Bioscience Engineering, TU Wien, Vienna, Austria; University of California, Riverside

## Abstract

In this work, we present the whole-genome sequence and the complete mitochondrial sequence of the black yeast-like strain Aureobasidium pullulans var. *aubasidani* CBS 100524, which produces the exopolysaccharide aubasidan and was previously isolated from *Betula* sp. slime flux from the Leningrad Region of Russia.

## Abstract (precis)

In this work, we present the whole-genome sequence and the complete mitochondrial sequence of the black yeast-like strain Aureobasidium pullulans var. *aubasidani* CBS 100524, which produces the exopolysaccharide aubasidan and was previously isolated from *Betula* sp. slime flux from the Leningrad region, Russia.

## ANNOUNCEMENT

Aureobasidium pullulans is a yeast-like ascomycete with industrial relevance due to its extracellular polysaccharides (1). The main exopolysaccharide of *A. pullulans* var. *aubasidani* strain CBS 100524 is aubasidan rather than pullulan (2, 3). This strain was previously isolated from plant exudates of a *Betula* sp. from the Leningrad Region of Russia (2). Despite the difference in the secreted extracellular polysaccharides, *A. pullulans* var. *aubasidani* strain CBS 100524 is part of a main phylogenetic group (phylogenetic difference below 0.25 based on a multilocus alignment with a bootstrap value of 100) within the *A. pullulans* species complex. This group also includes the ex-neotype strain *A. pullulans var. pullulans* CBS 584.75 and the sequenced strain *A. pullulans* var. *pullulans* EXF-150 (3).

*A. pullulans* strain CBS 100524 was cultivated in malt extract medium (30 g/liter malt extract, 1 g/liter peptone) at 24°C and 220 rpm for 24 h. The biomass was filtered through Miracloth (EMD Millipore Corp., Burlington, MA, USA), lyophilized, and stored at −20°C. Genomic DNA was extracted as described in reference 4, sheared through sonication, purified using the GeneJET PCR purification kit (Thermo Fisher Scientific, Inc., Waltham, MA, USA), and then size selected for 800-bp fragments using NEBNext Ultra sample purification beads (New England Biolabs, Ipswich, MA, USA). The library was prepared using the NEBNext Ultra II DNA library kit with purification beads and NEBNext multiplex oligos for Illumina (index primer set 2) (both New England Biolabs) and sequenced on a MiSeq instrument using a v3 reagent kit (600 cycles, 2 × 300-bp paired-end reads) (both Illumina, Inc., San Diego, CA, USA).

The sequencing yielded 2,892,731 read pairs. First, a crude *de novo* assembly was performed using SPAdes v3.13.1 (5) with default parameters. From this initial assembly, mitochondrial sequences were identified by a BLAST analysis against the nonredundant nucleotide database (6). Next, these sequences were used as seed input for NOVOplasty v3.7 (7) for a *de novo* assembly of the mitochondrial genome sequence (one circular contig; size, 37,556 bp; coverage, 358×). Using the mitochondrial genome sequence as index built with Bowtie v1.2.2 (8), the mitochondrial reads were extracted from the raw reads. The mitochondrion-free reads were then re-paired using Fastq-pair (9), quality checked and trimmed using Trimmomatic (10), leaving 2,543,186 read pairs, and then mapped against the reference genome *A. pullulans* strain EXF-150 (GenBank accession no. GCA_000721785.1) with BWA (11) and combined and sorted using SAMtools v1.7 (12) and Picard (13). A first genome representation was extracted using ANGSD v0.925 (Analysis of Next Generation Sequencing Data) (14). The genome assembly was iteratively improved using SSPACE-Standard v3.0 (15), GapFiller v1-10 (16), and Pilon v1.21 (17). tRNA genes were detected using tRNAscan-SE v1.3.1 (18). Genes were predicted with AUGUSTUS v3.3.2 (19), trained with the reference genome *A. pullulans* strain EXF-150 according to reference 20. The assembly was masked using RepeatMasker v4.0.9 (21), based on the Dfam_3.0 database to identify repetitive elements. We used QUAST v5.0.2 (22, 23), including the fungal (fungi_odb9) Benchmarking Universal Single-Copy Orthologs (BUSCO) v3.0.2 (24), for the final evaluation.

The assembly consists of 83 scaffolds (total sequence length, 30,265,078 bp; *N*_50_, 1,201,293 bp; GC content, 50.50%; mean coverage, 28×), and 10,978 genes (99.31% complete BUSCO genes found) and 353 tRNAs were predicted.

### Data availability.

The raw reads were uploaded to the Sequence Read Archive (SRA) under the accession no. SRR12830835. The complete genome sequence was deposited at DDBJ/ENA/GenBank under the accession no. JADGIM000000000. The version described in this paper is version JADGIM000000000.1. The complete mitochondrial genome sequence was deposited under GenBank accession no. MW148763.
